# Effects of patients’ hospital discharge preferences on uptake of clinical decision support

**DOI:** 10.1371/journal.pone.0247270

**Published:** 2021-03-08

**Authors:** James C. Cox, Ira L. Leeds, Vjollca Sadiraj, Kurt E. Schnier, John F. Sweeney

**Affiliations:** 1 Department of Economics and Experimental Economics Center, Georgia State University, Atlanta, Georgia, United States of America; 2 Department of Surgery, Johns Hopkins University School of Medicine, Baltimore, Maryland, United States of America; 3 Department of Economics and Business Management, University of California – Merced, Merced, California, United States of America; 4 Department of Surgery, Emory University School of Medicine, Atlanta, Georgia, United States of America; Brown University, UNITED STATES

## Abstract

The Centers for Medicare and Medicaid Services identified unplanned hospital readmissions as a critical healthcare quality and cost problem. Improvements in hospital discharge decision-making and post-discharge care are needed to address the problem. Utilization of clinical decision support (CDS) can improve discharge decision-making but little is known about the empirical significance of two opposing problems that can occur: (1) negligible uptake of CDS by providers or (2) over-reliance on CDS and underuse of other information. This paper reports an experiment where, in addition to electronic medical records (EMR), clinical decision-makers are provided subjective reports by standardized patients, or CDS information, or both. Subjective information, reports of being eager or reluctant for discharge, was obtained during examinations of standardized patients, who are regularly employed in medical education, and in our experiment had been given scripts for the experimental treatments. The CDS tool presents discharge recommendations obtained from econometric analysis of data from de-identified EMR of hospital patients. 38 clinical decision-makers in the experiment, who were third and fourth year medical students, discharged eight simulated patient encounters with an average length of stay 8.1 in the CDS supported group and 8.8 days in the control group. When the recommendation was “Discharge,” CDS uptake of “Discharge” recommendation was 20% higher for eager than reluctant patients. Compared to discharge decisions in the absence of patient reports: (i) odds of discharging reluctant standardized patients were 67% lower in the CDS-assisted group and 40% lower in the control (no-CDS) group; whereas (ii) odds of discharging eager standardized patients were 75% higher in the control group and similar in CDS-assisted group. These findings indicate that participants were neither ignoring nor over-relying on CDS.

## Introduction

Historically, the Centers for Medicare and Medicaid Services (CMS) has incurred over $17.5 billion in additional hospital charges annually from the 10–20% of its covered patients with unplanned hospital readmission within 30 days after discharge [1]. For the total U.S. inpatient population, the costs of hospital readmissions is over $41 billion annually [2]. The rate of unplanned readmissions is a metric for low quality healthcare as well as a cost inflator [3]. As a result, CMS has penalized hospitals with higher-than-expected readmission rates [4].

One of the most direct opportunities to reduce hospital readmission rates is to increase patients’ hospital length of stay (LOS) [5, 6], given that more than 30% of readmissions occur within a week after discharge [7]. However, increasing the average LOS overburdens health systems, worsening access to care and increasing total healthcare costs [8]. Therefore, a more targeted option for reducing hospital readmission rates is to prioritize discharging of patients that are most likely to avoid readmission, and vice versa [8, 9]. Clinical decision-support (CDS) tools may offer a low-resource and high-quality selection mechanism [10–12]. Such an approach may be particularly beneficial to surgical patients who exhibit well-established risks for readmission. Specifically, reported inconsistencies among surgeons’ stated discharge criteria, algorithmic estimates of their actual discharge criteria [13], and empirical criteria that predict unplanned readmissions suggest that discharge decision-making can be improved by application of evidence-based discharge criteria at the point of care [9, 14].

Application of complex but existing knowledge may be most easily facilitated by a data-driven CDS tool. Historically, CDS tools have been difficult to implement and inefficient for real-time use [15–18]. We have previously reported laboratory experiments in which subjects’ uptake of CDS patient discharge selection criteria improved discharge decision-making [9, 13, 19]. However, these prior studies did not incorporate how patient-clinician interactions may affect this decision-making process.

An open question is whether providers can integrate CDS objective information with subjective information obtained from examining patients to arrive at better discharge decisions that decrease length of stay *and* readmission rate. This question is central because of two opposing problems that can occur with *any* CDS tool: (1) there can be negligible uptake of the CDS recommendations by providers; or (2) the providers can be over-reliant on the CDS recommendations and underuse other information including subjective reports by patients. The purpose of this study is to use a behavioral experiment with clinician decision-makers and standardized patients to investigate the impact of human interaction on the discharge decision and the uptake of recommendations provided by the CDS tool. Results from the experiment will provide additional information relevant to decisions about deploying CDS on patient wards.

## Methods

Medical students were recruited for a 2×2 incentivized behavioral experiment with standardized patients to assess discharge practices following simulated surgical encounters with and without a decision-support tool and when interacting with different patient discharge preferences (“Eager” versus “Reluctant”). The multivariable treatment effects of decision-support and patient discharge preferences were assessed for (1) length of stay using least squares estimator and (2) likelihood of readmission, as a measure of quality of discharge decisions, using logit estimator. Differential effects of patient discharge preferences on concordance rates between decision-support recommendations and participants’ discharge decisions were assessed using *t*-test. Finally, discharge decisions in this standardized patient experiment were compared to the authors’ prior experiment [9] without standardized patients to assess effects of patient preferences on discharge decisions using logit estimator. In all regressions standard errors are clustered at participant level.

### Study design

**Fig 1:**
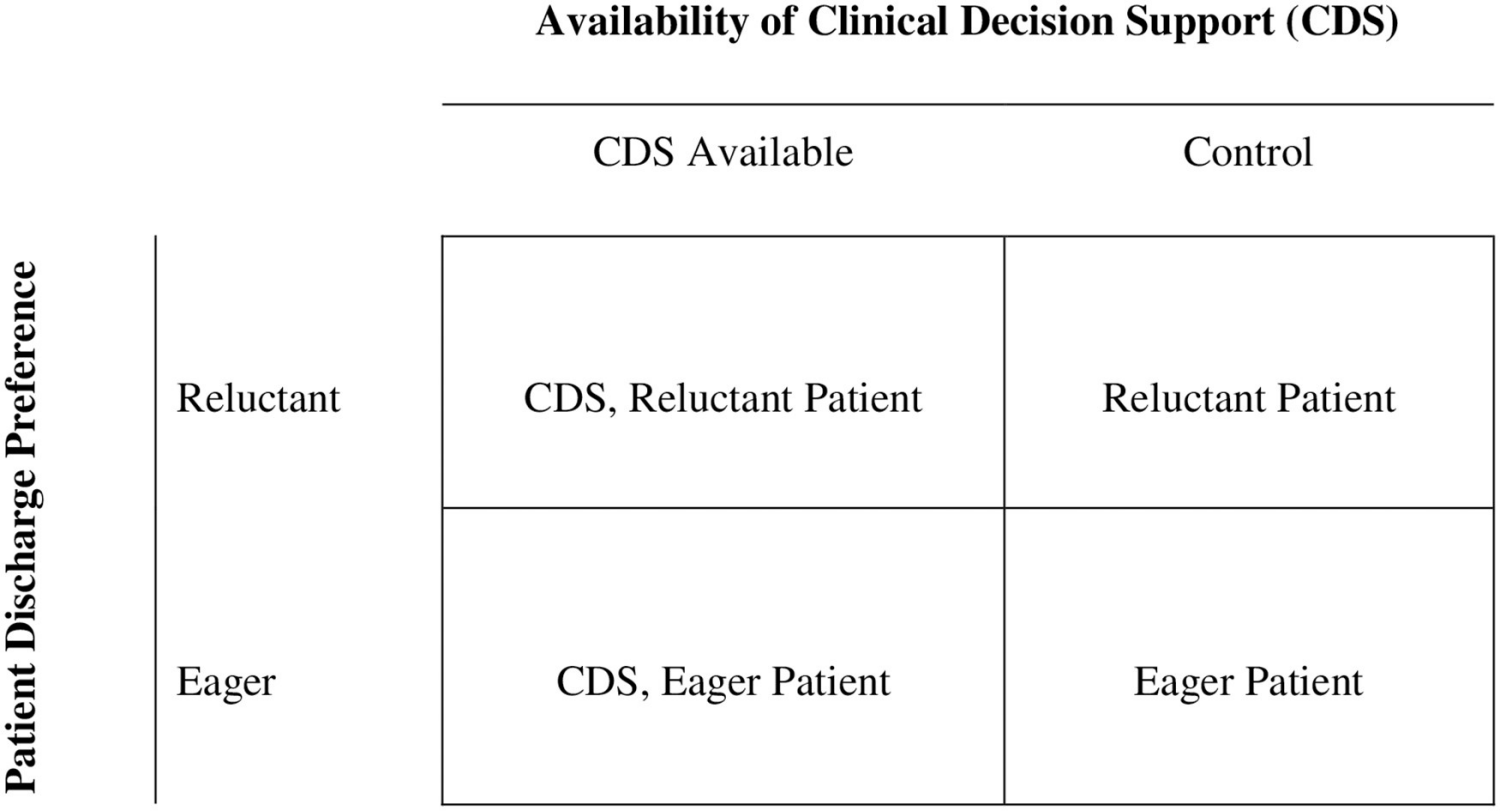
2×2 Behavioral experimental design. Note. Four groups of participants randomly assigned to whether they had the availability of a decision support tool and for each encounter with a patient eager or reluctant for discharge.

We conducted a 2x2 behavioral experiment comparing bedside clinician decision-makers with and without a CDS tool examining patients who are Eager versus Reluctant for discharge as shown in Fig 1.

Because these interactions are difficult to measure with real patient encounters, this form of behavioral study favored a simulated decision-making environment. Therefore, we recruited medical students to engage with a simulated hospital patient ward with standardized patients and using a medical school’s mock examination rooms traditionally used for the teaching curriculum’s Objective Structured Clinical Examinations (OSCE). We designed the experimental sessions to last approximately 2.5 hours. This study was approved by the Emory University Institutional Review Board and the Georgia State University Institutional Review Board. Written consent was obtained from participants.

### Experimental subjects

We recruited third and fourth year medical students to model clinician decision-making. They were the human subjects who made the discharge decisions in the experiment. All of these participants had prior experience interacting with standardized patients within the medical school’s OSCE program. Instructions provided to the subjects are contained in S1 Appendix.

### Electronic medical records

We selected eight patients from de-identified electronic medical records for general surgery patients from the “data warehouse” of a large southeastern teaching hospital, whose procedures were from the upper two-thirds of readmission risk (i.e., greater than 10% readmission risk) for all hospital stays. These eight electronic medical records were used in the experiment. Information was presented to subjects in the experiment with facsimiles of the (electronic) pages in the hospital’s electronic medical records system for these eight patients.

### Standardized patients

A cadre of around 100 skilled professionals who are trained to present clinical scenarios work at OSCE. These professionals present clinical scenarios in a standardized fashion, thus earning the title of “standardized patients.” Each standardized patient was matched with the de-identified electronic medical record of a distinct real patient included in the sample of eight real patients’ records. Each standardized patient was given enough information about the specific illness and course of treatment of the real patient they would portray to serve as a proxy for the real patient in an OSCE examination room (see example in S2 Appendix).

### Clinical decision support

Some treatments in the experiment included use of a clinical decision support (CDS) tool that presents evidence-based discharge recommendations obtained from econometric analysis of data from de-identified electronic health records of hospital patients. The estimated model and visualization of the CDS have been previously described [9, 19]. The model includes a dynamically updated daily probability of readmission within 30 days of discharge for a specific patient using clinical, demographic, and census data. The prediction model was developed from probit estimation with data for 3,202 general surgery patients. The data included observations of whether a patient was readmitted with the same diagnosis code within 30 days of discharge (the Medicare horizon), values of clinical variables during a patient’s stay, the duration of time spent outside and within the normal range of values expected for a particular clinical variable, counts of medications, images and transfusions, as well as a full set of interaction terms between the laboratory test and vital sign variables. The HIPAA “Safe Harbor” method was used to link medical records with census track data to obtain demographic data.

The CDS provides a statistically informed answer to the central question: “If this patient is discharged today, what is the likelihood of unplanned readmission within 30 days?” In this way, a current discharge decision can be informed by the aggregated experience with thousands of similar patients with known histories from the same institution. To make daily discharge recommendations, the CDS compares readmission likelihoods to procedure-specific target rates of readmission. These target rates are 10% reductions from procedure-specific historical readmission rates, a goal stated by the Center for Medicare and Medicaid Services in 2010. On a given day, the CDS recommends “Do Not Discharge Patient” if the point estimate of readmission likelihood exceeds the target readmission rate. If the target rate is between the point estimate and the upper bound of 80% confidence interval, the CDS reports “Physician Judgment.” If the upper bound of the 80% confidence interval is below the target rate, the CDS recommendation is “Discharge Patient.” This conservative criterion reflects choice of an estimated 10% error rate for the positive discharge recommendation.

### Experimental treatments

In one session, a specific standardized patient portraying a specific real patient would be instructed to follow the script to present herself as Eager to go home. In another session with different clinician decision-makers, the same standardized patient portraying the same real patient would be instructed to follow the other script to present herself as Reluctant to go home. Subjective standardized patient instructions varied between two sessions but the EMRs (and CDS when included) were identical. See S3 Appendix for an example of Eager and Reluctant standardized patient instructions. The clinician decision-makers all had complete access to the clinical variables for each case and participated in an exam encounter with the standardized patient. All decision-makers encountered four Eager and four Reluctant standardized patients.

The clinician decision-makers were randomized to treatments with a clinical decision-support tool with discharge recommendations (“CDS Supported”) or treatments without CDS (“Control”). The protocol of the experiment was organized into “experimental days” that paired 24 hour average values of clinical variables in a patient’s EMR with rounds of examining the standardized patients. In the first experimental day, each decision-maker examined all eight standardized patients and viewed the paired days’ EMRs. In experimental day 1, the paired day’s EMR was not necessarily calendar day 1 of hospital stay; it was, instead, randomly selected to be between one and two days before the discharge model would first recommend that the patient be discharged. This was done to avoid repeating multiple rounds of interaction with standardized patients in which no reasonable discharge could be contemplated. In subsequent experimental days the clinician decision-makers were rotated through encounters with each patient they had not yet discharged and viewed subsequent days’ paired EMRs. Because clinical decision-makers made discharge decisions independently, on experimental days after the first day different decision-makers could be examining different numbers of patients. An experimental session ended when every decision-maker had discharged all eight patients. The median of the distribution of total number of experimental days to discharge all eight patients is 7 (lower and upper quartiles are 6 and 8). The median total number of (“yes” and “no”) discharge decisions is 30.5 (lower and upper quartiles are 25 and 36). The operations for each patient encounter focused on intensive abdominal surgical procedures including: complex hepatobiliary reconstruction, pancreas resection, palliative gastrojejunostomy, pelvic exenteration, and colectomy. Participants’ order in the rotation of encounters was randomly determined. Each clinician decision-maker would first review EMR information and (in some treatments) CDS output updated to the current experimental day using a laptop computer outside the OSCE examination room. The participant would then enter the examination room to interview the patient, perform an exam, and after that enter his or her decision into the laptop of whether to discharge the patient on that experimental day. Whether each standardized patient was ultimately readmitted was determined by a random draw from a binomial distribution of the probit point estimate of readmission probability for the discharge day. To incentivize discharge-motivated clinician behaviors, each discharged patient that was not readmitted generated a $15 payment to the clinician decision-maker ($120 maximum possible payout for eight patients). Participants’ financial disincentive for prematurely discharging a patient was the increased probability of forgoing the $15 for a successful discharge (one that did not result in readmission).

### Other sources

To further study human-machine interactions, we compare discharge data from the 2×2 experiment with standardized patients reported herein to discharge data from a previously-reported laboratory experiment without standardized patients [9]. Since the EMR data for the eight patients used in the OSCE experiment with standardized patients were a subset of data used in the previous experiment without standardized patients, comparison of discharge decision responses for these eight patients between the two experiments provides additional insight into the effects of subjective information on quality of discharges and the uptake of evidence-based discharge criteria from CDS.

The experiment reported in [9] did not include standardized patients. It was conducted in a computer laboratory, not in the OCSE facility used for the experiment reported herein. As with the experiment reported herein and described above, the experiment in [9] included treatments with information provided by the CDS and facsimiles of EMR as well as treatments only with EMR information. Participants in the experiment reported in [9] were residents and third and fourth year medical students. They made discharge decisions for 30 “virtual” patients characterized by their electronic medical records. Discharge decisions made by subjects in both experiments using the same eight EMRs are pooled only for the analysis reported below in Table 4.

### Variables

For each patient encounter, we recorded whether the patient was discharged, the patient’s length of stay, and whether the patient was readmitted. For each decision-maker participant, we collected self-reported demographic information including gender, medical school GPA, undergraduate GPA, musical background, athletic background, and risk attitudes.

### Statistical analysis

Stata, version 16.0 is used for all data analysis. Participants’ demographics in the CDS Supported and Control groups were statistically compared using Fisher’s exact test. We use least squares regression with robust standard errors clustered at the participant level to estimate two individual treatment effects (1: decision support available; 2: information on patient discharge preference) and other determinants of length of hospital stay, such as whether patients’ procedure is from the High Risk group (defined by the historical readmission rate exceeding 0.17), the Start Date (the first examination day) and, for robustness check in one of the model specifications, several dummies of demographic variables (summary statistics reported in S4 Appendix). For the quality of discharge decisions, we use logistic regressions (with robust standard errors clustered at the participant level) to estimate the two individual treatment effects and other determinants (log of length of hospital stay, High Risk procedure, demographic covariates for robustness check) of the likelihood of a patient being readmitted. To estimate the differential effects of patient discharge preference and type of CDS recommendation on CDS uptake, we use *t*-tests to compare participants’ compliance rates when CDS recommendation is Discharge Patient. CDS recommendations for most patients on most experimental days in the standardized patient experiment were Physician Judgment or Discharge Patient. CDS recommendation is Do Not Discharge Patient only for one patient on the first examination date, so we use Fisher’s exact test (as compliance rate for each subject is either 1 or 0). Finally, we expand the data set from the field experiment herein with data from a laboratory experiment reported in [9] to estimate the effect of patient discharge preference (Reluctant or none, Eager or none) on participants’ discharge decisions using logistic regressions (with robust standard errors clustered at the participant level).

## Results

We recruited 38 clinical decision-maker participants for the experiment. Participants (or experimental subjects) were statistically more likely to be female (p = 0.049, Fisher’s exact test) in the Control (no-CDS) group while all other demographic variables were no different (S5 Appendix).

### Length of hospital stay

The average length of stay is 8.06 days in the CDS Supported group versus 8.83 days in the Control (no CDS) group suggesting a CDS effect of reduced length of stay by approximately 0.77 day (p = 0.015, two-sample *t*-test, t-stat = 2.56). Potential determinants of hospital length of stay include how long the patient has been in the hospital before experimental day 1 (Start Date), whether patient diagnostic procedure is from procedures with historical readmission risk exceeding 17% (High Risk), whether decisions are CDS-assisted, the interaction of the latter two dummies, and patient aversion to being discharged (Reluctant). Decisions on when to discharge a patient may also be affected by individual decision-maker characteristics captured in exceeding median GPA (proficiency), musical training [20], athletic training [21], risk aversion [22] and gender [23]. Table 1 reports least squares estimates of the determinants of length of stay, for model specifications with and without demographic covariates. The least squares estimate of CDS effect is about one day less in hospital stay (p-value < 0.02), Reluctant patients were kept about 2 days longer (p-value < 0.001) in the hospital than Eager patients, and High Risk procedure patients were kept about 0.8 days longer (p < 0.03). By the experimental design, it is not feasible for participants to discharge some patients earlier than the EMR record date used in the first experimental day. To address this concern, we also utilized censored regression that models the propensity to discharge patients on the very first day of examination. Following standard terminology, such observations are considered to be “left-censored” and, in our context, are interpreted as the participant could have discharged some patients earlier than the EMR record date used in the first experimental day. We observe 42 left-censored observations out of a total of 304. Censored-regression estimates are similar to the least squares estimates reported in Table 1. For censored regression model specification without demographic covariates, the estimated coefficient for “Reluctant” patient is 2.45 (95% CI is (1.85, 3.06), two-sided p-value < 0.001) and for “CDS Supported” is -1.03 (95% CI is (-1.84, -0.21), two-sided p-value = 0.014). These estimates are robust to adding demographic covariates: the estimated coefficient for “Discharge Reluctant” patient is 2.46 (95% CI is (1.85, 3.06), two-sided p-value<0.001) and for “CDS Supported” is -1.23 (95% CI is (-2.10, -0.36), two-sided p-value = 0.006). We conclude that the estimated treatment effects reported in Table 1 are robust to left-censoring.

**Table 1:** Linear regression (OLS) models of length of stay on decision-support availability, degree of readmission risk, and patient preference for discharge.

	No (demographic) Covariates			With (demographic) Covariates		
Variables	Coefficient	(95% CI)	P-value	Coefficient	(95% CI)	P-value
Start Date	0.65	(0.53, 0.77)	0.000	0.65	(0.53, 0.77)	0.000
High Risk	0.81	(0.14, 1.48)	0.019	0.81	(0.13, 1.49)	0.021
CDS Supported	-0.96	(-1.73, -0.20)	0.015	-1.16	(-1.99, -0.33)	0.008
CDS + High Risk	0.31	(-0.36, 0.98)	0.352	0.31	(-0.37, 0.99)	0.357
Reluctant	2.10	(1.52, 2.68)	0.000	2.10	(1.52, 2.69)	0.000
Constant	3.69	(2.80, 4.57)	0.000	3.79	(2.78, 4.80)	0.000
R-Squared	0.587			0.597		

Notes. The dependent variable is the length of hospital stay. Reference category is no-CDS-supported decisions of Eager patients from no-High-Risk (procedure) group. Total number of observations is 304. Number of subjects is 38. 95% CI and (two-sided) p-values are for robust standard errors clustered at the participant level. Median GPA for Medical School is 3.5 and for Undergrad studies is 3.7. Demographic Covariates not shown include dummies for Undergrad GPA>3.7(median), Medical School GPA>3.5(median), Female, Musical training, Athletic training, and Risk Attitudes. Five patients (out of eight patients) were from procedures with historical readmission rates exceeding 17%. High Risk is a dummy variable that takes value 1 for these five patients and 0 for the remaining patients.

### Quality of discharge

An important metric of the quality of discharge is whether a discharged patient is readmitted. We use logistic regression to infer the effect of CDS on the risk of unplanned readmission. Other determinants include length of hospital stay, whether patient is from the High Risk (procedure) group, and the interaction of High Risk with availability of CDS. In Table 2, the reference category is no-CDS-supported decisions for Eager patients from no-High-Risk procedure group. Longer lengths of stay were protective against readmission (p < 0.001). For patients deemed High Risk for being readmitted, compared to no High Risk category odds of readmission indeed triple (3.30, p<0.02) in the Control treatment, but for CDS-assisted decisions, there was a borderline significant (p < 0.09) effect of 31% (= 1–0.21x3.30) decrease in odds of readmission. Our data indicate that the quality of discharge is not affected by patient preference for discharge; the estimated Odds Ratio for a Reluctant patient is not statistically significant (p > 0.96). These estimates are robust to inclusion of demographic covariates.

**Table 2:** Multivariable logistic regressions of readmission risk on decision-support availability, degree of readmission risk, and patient preference for discharge.

	No (demographic) Covariates			With (demographic) Covariates		
Variables	Odds Ratio	(95% CI)	P-value	Odds Ratio	(95% CI)	P-value
Length of Stay†	0.06	(0.01, 0.28)	0.000	0.07	(0.02, 0.31)	0.000
High Risk	3.30	(1.28, 8.54)	0.014	3.31	(1.22, 9.00)	0.019
CDS Supported	1.10	(0.37, 3.30)	0.861	1.11	(0.37, 3.33)	0.857
High Risk+ CDS	0.21	(0.04, 1.27)	0.090	0.20	(0.03, 1.24)	0.084
Reluctant	1.01	(0.37, 2.73)	0.984	1.01	(0.35, 2.94)	0.985
Pseudo R^2^	0.130			0.165		

Notes. Dependent variable is a binary variable that takes value 1 if a patient is readmitted after being discharged. Reference category is no-CDS-supported decisions of Eager patients from no-High-Risk (procedure) group. Total number observations is 304. Number of subjects is 38. Median GPA for Medical School is 3.5 and for Undergrad studies is 3.7. Demographic Covariates not shown include dummies for Undergrad GPA > 3.7 (median), Medical School GPA > 3.5 (median), Female, Musical training, Athletic training, and Risk Attitudes. Five patients (out of eight patients) were from procedures with historical readmission rates exceeding 17%. High Risk is a dummy variable that takes value 1 for these patients and 0 for the remaining patients. 95% CI and two-sided p-values are for robust standard errors clustered at the participant level.

† Natural logarithmic transformation for better fit.

### Concordance rates

Recall that in the Control treatment participants were making discharge decisions based on daily EMR information displayed on their laptop screens and examinations of standardized patients. In the CDS treatment, participants were provided with additional information that included the predicted probability of readmission (with 80% confidence interval) if the patient were to be discharged on each experimental day up to the present day. The CDS presented dynamically-updated daily discharge recommendations based on procedure-specific target readmission rates that were 10% improvements on historically-observed rates. There were three types of recommendations: “Do Not Discharge Patient” if the predicted readmission probability was above the target rate; “Physician Judgment” if the target rate was between the predicted readmission probability and the upper bound of the 80% confidence interval; or “Discharge Patient” if the upper bound of the confidence interval was below the target rate.

To infer CDS uptake we looked at agreement rates between actual participant decisions and CDS recommendations of Do Not Discharge Patient or Discharge Patient. When participants see no recommendations (or estimated readmission probabilities), their decisions can nevertheless be the same as the CDS recommendation because both experiment decision-makers and the prediction model use patients’ daily information from EMRs. The main difference rests in experiment participants’ decisions reflecting their own individual subjective readmission likelihood whereas the CDS recommendations (statistically) aggregate physicians-of-record subjective readmission likelihoods and calibrate them to the success of discharge measured by readmissions of real patients. We constructed a new variable, consistency rate, that takes its values as the fraction of time the participants’ decisions were the same as the CDS recommendation, Discharge Patient or Do Not Discharge Patient separately for Eager and Reluctant standardized patients. We observe a higher overall concordance in the CDS supported group (63% versus 59% in the Control) but the two figures are not statistically different (two sample t-test, p = 0.466). Agreement rates were substantially lower when the CDS recommendation was Discharge Patient, likely reflecting the inherent risk aversion of discharge decision-makers. CDS recommendation was Do Not Discharge Patient for only the first examination day of one patient, so we have 38 observations. With the exception of one subject examining an Eager type of this patient, all participants (CDS-assisted or not) did not discharge the patient of either type (Reluctant or Eager) on the first day of examination. CDS makes recommendation Discharge Patient for all eight patients, one or two days after the first day of examination. We have a total of 816 observations. Since there is more than one observation per subject, to ensure independence of observations we generated the average consistency rate separately for each subject for each patient type (Reluctant, Eager). The new variable takes values in the (0.14, 1] interval rather than being binary. The net treatment effect of CDS for Reluctant+Eager patients is 10% increase (two-sample t-test, p = 0.083) in concordance with the increased rate being more pronounced in encounters with Reluctant patients (12%, two-sample t-test, p = 0.019) than with Eager patients (7%, two-sample t-test, p = 0.410). Table 3 reports the summary results of concordance. In CDS-assisted decisions, there is a 20 percentage point higher concordance with Discharge Patient recommendation observed for Eager patients (58%) than for Reluctant patients (38%). This suggests that participants were responding to information conveyed by standardized patients and integrated patients’ stated readiness in their discharge decisions. Next, to shed some light on human-machine interactions, we look more closely at the patient preference effect on decisions.

**Table 3:** Concordance between decision-support recommendation and study participants’ decisions to discharge stratified by standardized patient preferences about discharge.

		Mean (95% CI)					
Treatment	Recommendation	Reluctant		Eager		Reluct.+ Eager	
**CDS Supported**	Discharge	0.38 (0.28, 0.47)		0.58 (0.45, 0.70)		0.48 (0.40, 0.56)	
	Do Not Discharge	100%		88.89%		94.74%	
	All Recommendations	0.59 (0.46, 0.72)		0.68 (0.56, 0.80)		0.63 (0.55, 0.72)	
**Control (no-CDS)**	Discharge	0.25 (0.21, 0.29)		0.51 (0.39, 0.63)		0.38 (0.31, 0.46)	
	Do Not Discharge	100%		100%		100%	
	All Recommendations	0.51 (0.37, 0.65)		0.67 (0.55, 0.79)		0.59 (0.50, 0.68)	
		**Diff.**	**P-value**	**Diff.**	**P-value**	**Diff.**	**P-value**
**Net CDS Effect**	Discharge	0.12	0.019	0.07	0.410	0.10	0.083
	Do Not Discharge		1.00		1.00		1.00
	All Recommendations	0.08	0.392	0.01	0.897	0.05	0.466

Notes. Table reports averages of subjects’ mean consistency rates across treatments. Consistency variable takes value 1 if subject’s discharge decision is the same as the CDS recommendation; discharge decisions on days for which CDS tool makes no recommendation are not included. For each subject, we created the mean consistency rate separately for each recommendation (Discharge, Do not Discharge) and for each patient type (Reluctant, Eager). Two-sided p-values in the Net CDS Effect part of the table are for the two-sample *t*-test in Discharge and All Recommendations rows, and for Fisher’s exact test in Do Not Discharge row.

### Standardized patient behavior effect

The data analysis reported in Table 4 provides an answer to one of the central questions of this paper: compared to decisions based on EMR, what effects do standardized patient reports of being Reluctant or Eager to be discharged have on discharge decision-making? In order to answer this question, we use data from the experiment with standardized patients (reported in this paper) together with data from an earlier experiment reported in [9] that did not include standardized patients. The econometric model uses dummy variables for standardized patients providing reports based on Eager or Reluctant scripts. We use participants’ decisions in the treatment without CDS in the experiment without standardized patients reported in [9] to categorize each day of a de-identified patient’s EMR, as follows. For each patient, we identify the first and fourth quartiles of length of hospital stay observed in that treatment (no-CDS, no standardized patients). Days before the first quartile day of discharge are classified as “Before First Quartile” whereas days after the fourth quartile of day of discharge are classified as “After Fourth Quartile.”

**Table 4:** Patient preference effects on discharge decisions according to logistic regression.

	CDS-assisted Decisions			No CDS-assisted Decisions		
Variables	Odds Ratio	95% CI	P-value	Odds Ratio	95% CI	P-value
**Reluctant Effect**						
Reluctant	0.33	(0.18, 0.60)	0.000	0.60	(0.39, 0.94)	0.025
Reluctant x After Fourth Quartile^a^	0.52	(0.18, 1.49)	0.226	1.05	(0.45, 2.46)	0.905
Pseudo R^2^ {Nobs, Clusters}	0.080 {753, 42}			0.117 {1077, 43}		
**Eager Effect**						
Eager	1.31	(0.65, 2.65)	0.448	1.75	(1.06, 2.91)	0.029
Eager x Before First Quartile^b^	0.55	(0.25, 1.22)	0.144	3.23	(1.45, 7.20)	0.004
Pseudo R^2^ {Nobs, Clusters}	0.126 {615, 42}			0.164 {893, 43}		

Notes. Dependent variable: An indicator variable that takes value 1 if the patient is discharged and 0 if the patient is kept in the hospital. Data from the experiment without standardized patients and the Reluctant standardized patient group were combined for the top three rows while data from the experiment without standardized patients and Eager patient group were combined for the bottom three rows. Treatment variable is a dummy variable for a standardized patient (Reluctant or Eager). Regressors include: a dummy variable for Before First Quartile or After Fourth Quartile and its interaction with Reluctant or Eager standardized patient. Median GPA for Medical School is 3.5 and for Undergrad studies is 3.7. Other (not shown) covariates are dummies for day of hospital stay (fixed effects), Undergrad GPA>3.7(median), Medical School GPA>3.5(median), Female, Musical training, Athletic training, Risk Attitudes. 95% CI and two-sided p-values are for robust standard errors clustered at the participant level.

^a^ “After Fourth Quartile” was categorized based on the fourth quartile of observed patients’ discharge day for the subset of data from the experiment without CDS and without standardized patients, as reported in Other Source section.

^b^ “Before First Quartile” was categorized based on the first quartile of observed patients’ discharge day for the subset of data from the experiment without CDS and without standardized patients, as reported in the Other Source section.

As reported above, Reluctant standardized patients are kept longer in the hospital than Eager standardized patients. While patient preference for discharge is an important signal of patient readiness for discharge, unnecessarily delayed discharge (of Reluctant patients) reduces others’ access to hospital care services while premature discharge (of Eager patients) adversely affects the quality of medical care.

Table 4 shows that patient discharge preferences can affect clinician decision-making with and without a decision-support tool. As shown in the first row of Table 4, compared to discharging a patient solely on EMR data, odds of discharging a Reluctant standardized patient decrease by 67% (= 1–0.33, p<0.001) when CDS assisted but by only 40% (= 1–0.60, p = 0.025) in the Control (no-CDS) treatment. As shown in the second row of Table 4, there is insignificant additional Reluctant patient effect on days the patient is in After Fourth Quartile. These estimates reveal that participants are cautious, and even more so when CDS supported, about discharging patients whose reports signal they do not feel ready to be discharged. On the other hand, compared to discharging a patient based solely on EMR data, discharge odds of an Eager standardized patient in the Control treatment (no-CDS) increase by 75% (= 1.75–1, p = 0.029) and when CDS assisted, by 31% (= 1.31–1), but the latter is not statistically significant (p = 0.448). If we look on early days in the hospital (classified as Before First Quartile), compared to discharges based only on EMR, the discharge odds of an Eager standardized patient more than quintuple (= 1.75x3.23) in the Control treatment (no CDS), which could adversely affect the quality of medical care. In CDS-assisted decisions, however, we don’t see such effect. These findings suggest that, absent CDS, patients with preferences for discharge may be prematurely discharged, and CDS offers a protection against this.

## Discussion

Even in well-regulated healthcare systems, variations in care exist that ultimately lead to disparities in outcomes [24]. Growing research in both large-scale administrative claims analyses as well as individual-oriented, behavioral studies suggest that the decisions clinicians make can drive variations in care that ultimately affect observed differences in outcomes [13, 25, 26]. Clinician decision-making for discharging patients can be characterized using the “bounded rationality” behavioral economic decision-making model described by Simon [27] for which he was ultimately awarded the Nobel Prize. Bounded rationality models presume that decision-making in complex situations is not limited by too little information but, instead, by too little capacity of the decision-maker to fully process the available troves of information when making a decision.

Even though Simon’s bounded rationality model was first described in 1955, modern healthcare fits his original construct well. Clinicians are deluged with both objective and subjective data, and the discharge decision represents a critical point that the individual clinician needs to get right. If discharged too early, patients face increased risk of readmission and possible harm. If discharged too late, scarce resources of the healthcare system are used unnecessarily. Dual process theory has arisen from Simon’s research as one of the best working frameworks for how clinicians handle crux decisions with imperfectly ascertainable information. Dual process theory suggests that two modes of analysis occur in the human decision-maker, System 1 (fast, heuristic thinking) and System 2 (slow, deliberative analysis) [28]. These two processes combine to facilitate decision-making in real-time that is both valid and efficient.

System 2 thinking is relatively easy to model and analyze. For many specialty-specific contexts, we know what clinical and socioeconomic predictors of patients drive readmissions [14, 29], and we have previously shown how these impact clinicians’ views and implicit behaviors with discharge decision-making [9, 13, 25]. However, we have a dearth of knowledge about how subjective bedside findings and patient behaviors influence the hard predictors of discharge decision-making and readmission. The influence of System 1 on discharge decision-making has had little formal study [30, 31], and none using behavioral experiments to control for external factors.

A central purpose of this study was to use behavioral experiments with clinician decision-makers and standardized patients to study whether providers can integrate CDS objective information with subjective information obtained from examining patients to arrive at better discharge decisions. Would decision-makers’ discharge choices reflect use of both CDS information and patient reports or would they neglect one source of information and over-rely on the other?

One-half of the subjects (i.e., clinician decision-makers) in the experiment participated in sessions using information from a facsimile of de-identified EMRs. The other one-half of the subjects participated in sessions using information from the CDS and the EMR facsimile. All subjects also received information from examining standardized patients.

Each of the standardized patients was given instructions for portraying an individual hospital patient with a specific illness and course of treatment. In addition, one-half of the standardized patients in each session were given instructions to report feeling well and being Eager to go home. The other one-half were given instructions to report feeling badly and being Reluctant to be discharged. Each subject encountered both Eager and Reluctant standardized patients in each session.

Results from the standardized patient experiment show uptake of the CDS recommendations was significantly affected by patient preferences about being discharged. Length of stay of Reluctant patients increased by 2 days (compared to Eager patients). There is an asymmetric uptake of the CDS recommendations, with participants being less willing to adopt Discharge Patient recommendations than Do Not Discharge Patient recommendations. The likelihood of patient discharge decreased significantly when the CDS recommendation was Discharge Patient but the standardized patient was randomized to Reluctant. Similarly, the likelihood that the patient was discharged was higher when a Discharge Patient CDS recommendation coincided with an Eager patient type than when it diverged from a Reluctant patient type. Participants were more likely to adhere to CDS recommendations when concordant with patient preferences but participants’ decisions reflected both patient discharge preferences and CDS discharge recommendations. Compared to decisions in the absence of standardized patients, odds of discharge of Reluctant patients were 67% lower for CDS-assisted decisions and 40% lower in the Control group. Absent CDS, the opposite pattern is observed for Eager patients, with odds of discharge increasing by 75%.

### Limitations

Limitations of this study include the intrinsic *ex vivo* nature of behavioral laboratory experiments and the small participant population. Behavioral laboratory experiments are useful when large-scale experiments in a real clinical environment are not practical. In the case of discharge decision-making, the heterogeneous nature of hospital discharges would mean the ongoing collection of hundreds of patients’ clinical data as well as their psychosocial preferences in order to observe how patient behavior affected discharge decision-making recommendations with and without CDS tools. In the particular institutional setting of this study, the role of this investigation was to help better understand these human-machine interactions prior to deployment. We have tried to mitigate any effects of a laboratory-based setting by appropriately incentivizing participants and using real clinical data that is comparable to what would be encountered by these decision-makers in their clinical roles.

### Next steps

A remaining open question is whether providers can integrate CDS objective information with subjective information obtained from examining patients on patient wards to arrive at better discharge decisions, which decrease length of stay *and* readmission rate. This question is central because of two opposing problems that can occur with *any* CDS: (1) there can be negligible uptake of the CDS by providers; or (2) the providers can be over-reliant on the CDS and underuse other information. The next step in testing the CDS will come from a field experiment in the form of an intervention on patient wards. Before that is possible, we are developing a beta version of the CDS that can interact with EMR in real time.

## Conclusion

This behavioral economic experimental study reveals that objective as well as subjective information about patients influences clinical decision-makers’ discharge decisions and how they respond to the recommendations of a clinical decision support tool. In addition, clinical decision-support may help warn against patients whose subjective preferences for discharge are inconsistent with objective clinical data.

## Supporting information

S1 Appendix Subject instructions. (PDF)

S2 Appendix Example of clinical information. (DOCX)

S3 Appendix Example of standardized patient scripts. (DOCX)

S4 Appendix Participant demographics. (DOCX)

S5 Appendix Raw data. (XLS)

S6 Appendix Stata code. (PDF)
