# Supplementary material for: Effects of patients’ hospital discharge preferences on uptake of clinical decision support
Source: PLoS One. 2021 Mar 8;16(3):e0247270. doi: 10.1371/journal.pone.0247270 (PMC7939268; doi:10.1371/journal.pone.0247270)
Supplement: S1 Appendix — (PDF) [file pone.0247270.s001.pdf]

Welcome

Team Members:

Dr. John F. Sweeney

Dr. James C. Cox

Dr. Kurt E. Schnier

Dr. Vjollca Sadiraj

Kevin Ackaramongkolrotn

Connie Coralli

Gina Shannon

Val Brown

William Knechtle

Main Idea

You are participating in a study about <u>post surgical discharge decisions</u>

Today you'll be <u>rounding</u> on **8** post surgical patients.

You'll be rounding on them "daily" until you have discharged everyone.

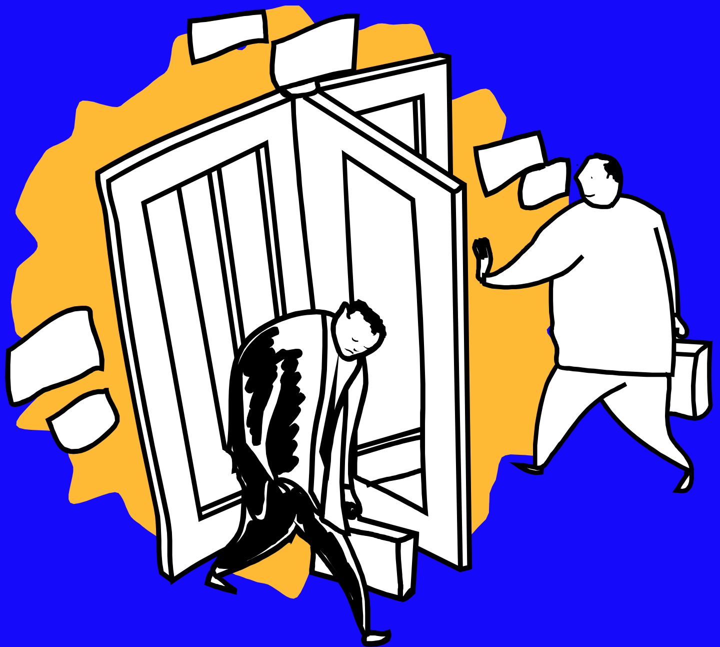

Schedule

- You will be given a list of room numbers which will show the order in which you are to rotate. You will be provided a laptop which you will carry with you during your rounds.

- There is an ID number on the top of your “rounding” schedule – If you want to get paid you’ll need to turn in this ID number at the end of the experiment.

Experiment.	*Your ID: #Z##Z#Z*
Schedule	
Room 1	
Room 6	
Room 5	
Room 7	
Room 2	
Room 4	
Room 3	
Room 8	

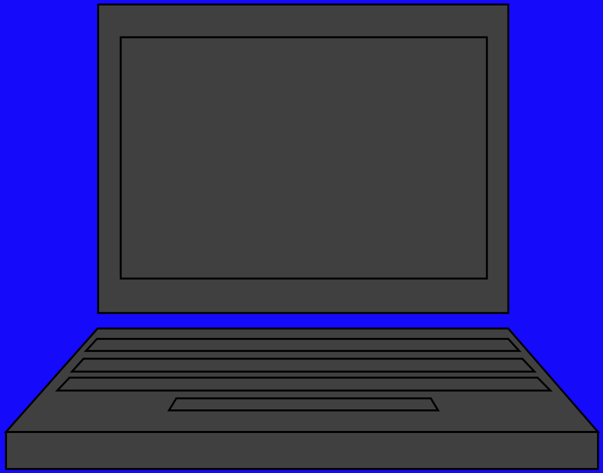

Schedule

Your first patient will be the one in the top row of your list.

1. Go to the room in the top row of your list
2. Look at the patient’s name on the door
3. Find that patient’s information in your computer.

Do not open the patient’s information until you are outside that patient’s door.

SCHEDULE EXAMPLE

Experiment.	Your ID: #Z##Z#Z
Schedule	
Room 1	
Room 6	
Room 5	
Room 7	
Room 2	
Room 4	
Room 3	
Room 8	

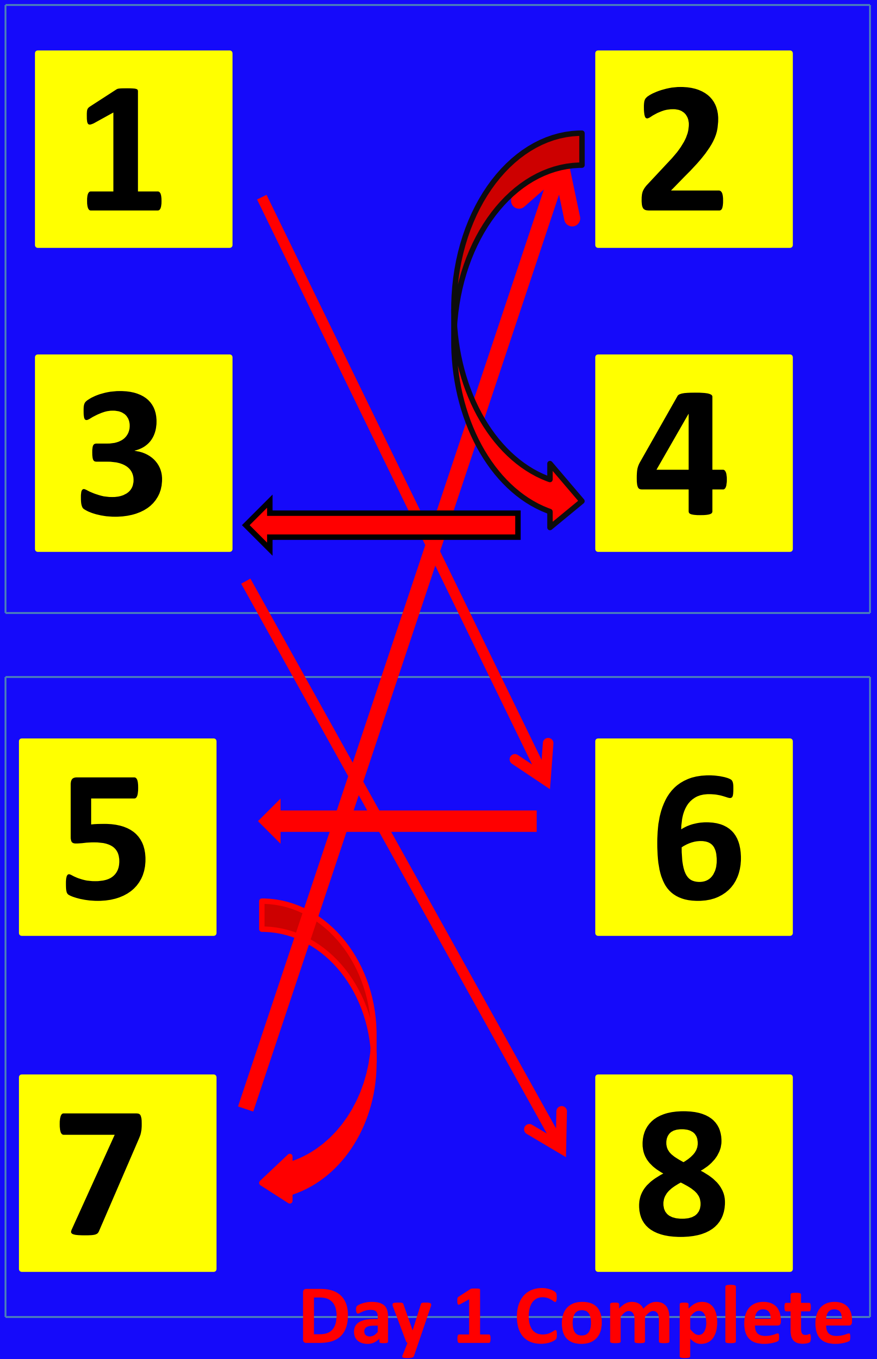

- On the laptop you will find various records for each of your patients.
- Some of them may contain "decision support" information which you may use or not as you see fit.
- The information you are provided from the records and patient may vary from one patient to another.
- The information each of you gets may vary one to another.
- Avoid allowing the behavior or other students to influence your behavior.

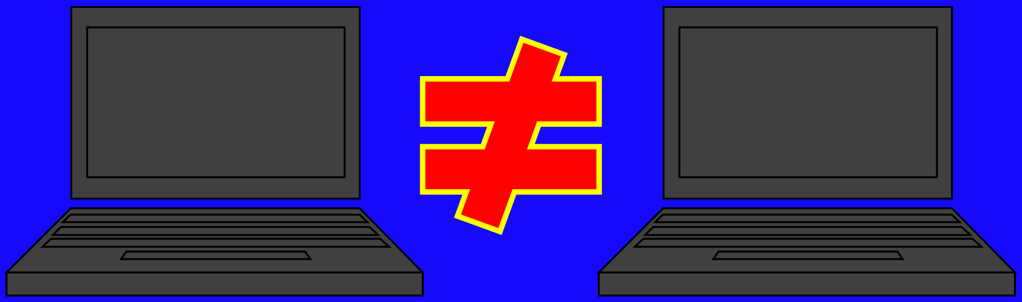

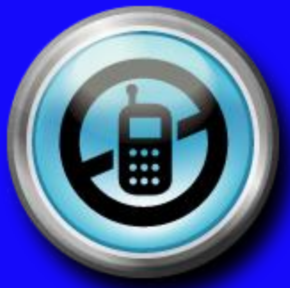

Rules

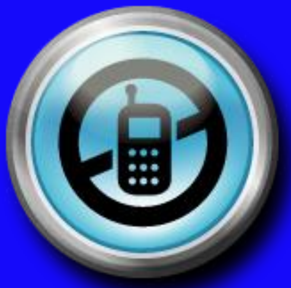

- No discussions about the cases or the structure of the experiment are allowed.

- No use of technology other than the computerized records is allowed. Your cell phones must be off. Please turn them off at the beginning of the experiment and do not turn them back on until the experiment is finished.

- You will be seeing standardized patients today and, as always, your interactions with them should be limited to those you would have with any other patient.

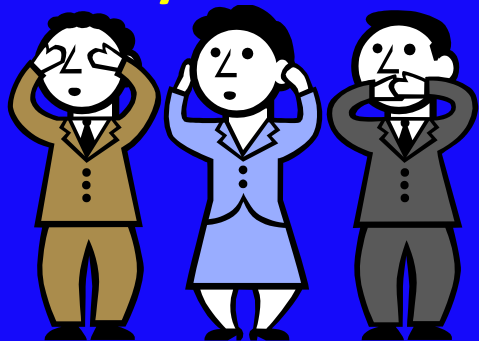

Rules
(continued)

- Do not inquire about what's happened with other students.

- Each of you has a list of patients that is unique and distinct. Please make no inferences about what or how other subjects are doing.

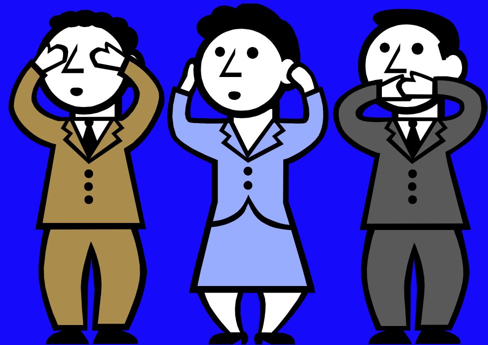

When you discharge a patient

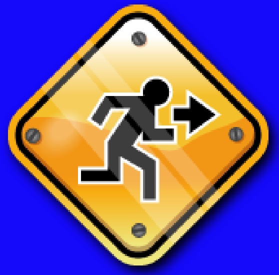

- The patient name will disappear from the patient list on your computer whether or not the discharge was successful.

- If you receive a message that a discharged patient was readmitted, please don't share this with the patients or other subjects.

- Nor should you make any inferences about the subsequent readmission as you will not be managing that admission.

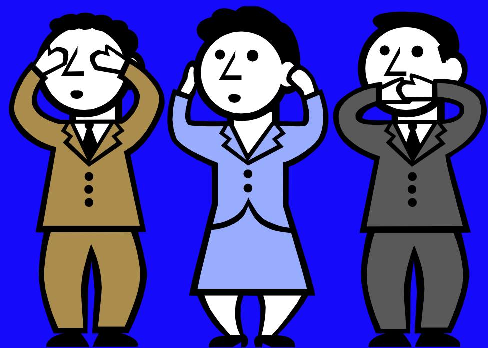

Money

- On each experimental day you'll be expected to make a decision to discharge or not to discharge. For each "successful" decision, i.e. for each discharged patient who's not readmitted, you'll be paid $15.

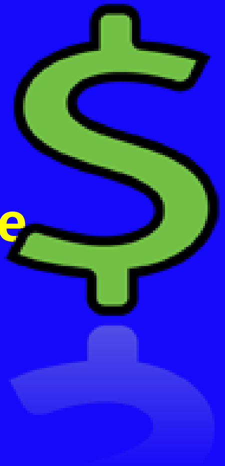

- You will not be paid for discharged patients who are readmitted.

- There is an ID number on the top of your "rounding" schedule – If you want to get paid you'll need to turn in this ID number at the end of the experiment.

Rooms

- If you are in Rooms 1-8 you'll be rotating in Suites 308 & 310.

- If you are in Rooms 9-16 you'll be rotating through Suites 320 & 322.

Pace

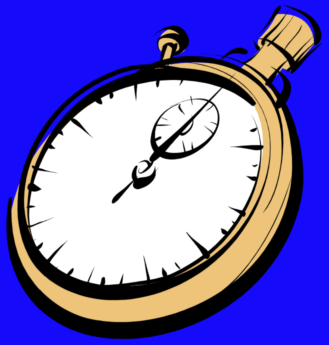

- This is a fast moving event.
- For the first experimental day you'll have 3 min. 45 sec. to review the records and see the patient and 15 sec. to rotate.
- For subsequent days, you'll have 2 min. 45 sec., again with 15 sec. to rotate.
- There will be a start and stop announcement for each patient encounter.
- Monitors in the suites will tell you when you are moving to a new experimental day.

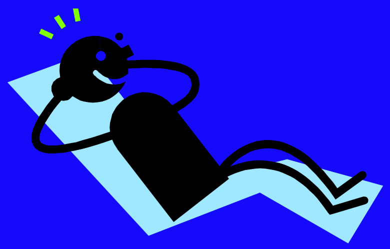

Break

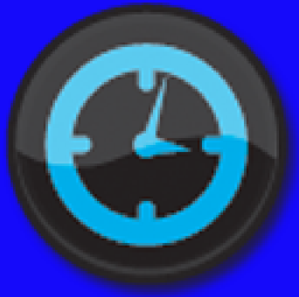

- There will be a 15 min. break around 3 PM. Some of you will spend the break waiting in the hallway – feel free to use the facilities if you need to.

- Others will be asked to spend the break in Room 351 – you may also use the facilities if you need to.

- A proctor will direct you as to which place you should stay during the break.

- *Avoid any discussion of the cases or experiment during the break.*

- <u>After</u> the break you may be given a different order for your rounds to follow.

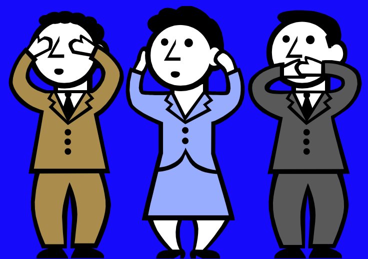

Questions ?
