# Supplementary material for: Effects of patients’ hospital discharge preferences on uptake of clinical decision support
Source: PLoS One. 2021 Mar 8;16(3):e0247270. doi: 10.1371/journal.pone.0247270 (PMC7939268; doi:10.1371/journal.pone.0247270)
Supplement: S2 Appendix — (DOCX) [file pone.0247270.s002.docx]

**S2 Appendix [Example of Clinical Information Given to Standardized Patients]**

Here is an example of the sketch of clinical information provided to the standardized patients. The table reports a summary of some of the clinical information in the EHR of the patient known as Ashley Barnes in the experiment. It shows the patient’s pseudonym, real age, and real surgical procedure. It also reports the experimental day, and real hospital stay day, pain score, pain medication, stool count, and type of diet.

**Example of Patient Clinical Information Given to Standardized Patients**

| **27780512 Barnes, Ashley COLECTOMY +/- COLOSTOMY** | | | | | | | | | | |  |
| --- | --- | --- | --- | --- | --- | --- | --- | --- | --- | --- | --- |
|  | This is a 28 year old female with a history of Peutz-Jegher's syndrome that underwent a left colectomy for treatment of a colon mass. She is now %d-days post op. | | | | | | | | | |  |
| Experiment Day | | 1 | 2 | 3 | 4 | 5 | 6 | 7 | 8 | 9 | |
| Hospital Day | | 1 | 2 | 3 | 4 | 5 | 6 | 7 | 8 | 9 | |
| Pain Score | | 0 | 0 | 5 | 0 | 3 | 4 | 6 | 0 | 0 | |
| Pain Med | |  |  |  |  |  |  |  | PO |  | |
| Stool Count | |  |  |  |  |  |  |  | 2 |  | |
| Diet | | NPO | NPO/Solids | Solids | NPO | NPO | NPO | NPO/Clear | Clear/Solids | Solids | |
