# Supplementary material for: Effects of patients’ hospital discharge preferences on uptake of clinical decision support
Source: PLoS One. 2021 Mar 8;16(3):e0247270. doi: 10.1371/journal.pone.0247270 (PMC7939268; doi:10.1371/journal.pone.0247270)
Supplement: S3 Appendix — (DOCX) [file pone.0247270.s003.docx]

**S3 Appendix [Scripts Given to Standardized Patients]**

Here shows the alternative scripts given to the actor playing “Ashley” (see Appendix 2) in the treatments in which Ashley was Reluctant or Eager to be discharged. The Reluctant and Eager treatments differ in the instructions for patient portrayal in the categories labeled appearance, pain score, activity, bowel function, diet, and social support. Note that, on days earlier than the seventh day in the hospital, reluctant behavior is consistent with the clinical data because Ashley’s diet is NPO (nothing by mouth) whereas on later days reluctant behavior might simply convey a general pessimistic attitude and stop serving as an informative signal of her health status.

**Scripts for Reluctant and Eager for Discharge Given to Standardized Patients**

|  | **Reluctant to go home** | **Eager to go home** |
| --- | --- | --- |
| **Appearance** | Looks disheveled and uncomfortable | Looks well and is asking to leave as soon as MD thinks it’s OK |
| **Pain Score** | Pain is minimally covered with current pain meds | Pain is tolerable and meds help control the discomfort |
| **Activity** | Having difficulty getting out of bed alone and has trouble walking in the hallways | Up and walking in the hallway; no problem getting out of bed |
| **Bowel Function** | Is having bowel function but still feels bloated and uncomfortable | Passing gas and moving bowels normally no problem there |
| **Diet** | Not interested in any of the food that is brought to them and overall no appetite; has nausea when eats | Tolerating food and has good appetite |
| **Social Support** | No one to pick the patient up until much later in the day; worried that he/she will be alone much of the day and therefore may have some problems | Plenty of home support; could leave right now if provider wants to send the patient home |
