# Supplementary material for: Effects of patients’ hospital discharge preferences on uptake of clinical decision support
Source: PLoS One. 2021 Mar 8;16(3):e0247270. doi: 10.1371/journal.pone.0247270 (PMC7939268; doi:10.1371/journal.pone.0247270)
Supplement: S4 Appendix — (DOCX) [file pone.0247270.s004.docx]

**S4 Appendix. Percentage of subjects whose response in the questionnaire is as in the first column**

| **Experiment** | **With Standardized Patients** CDS-Assisted^a^ Control^b^ p-value | | | **Without Standardized Patients**  CDS-Assisted^c^ Control p-value | | |
| --- | --- | --- | --- | --- | --- | --- |
|  |  |  |  |  |  |  |
| Medical School GPA > 3.5 | 26.32% | 31.58% | 1.000 | 21.74% | 33.33% | 0.517 |
| Undergrad GPA > 3.7 | 68.42% | 52.63% | 0.508 | 78.26% | 79.17% | 1.000 |
|  |  |  |  |  |  |  |
| Female | 21.05% | 52.63% | 0.091 | 65.22% | 45.83% | 0.244 |
| Athletic Training | 31.58% | 5.26% | 0.090 | 34.78% | 37.50% | 1.000 |
| Musical Training | 52.63% | 57.89% | 1.000 | 30.43% | 58.33% | 0.080 |
|  |  |  |  |  |  |  |
| Risk Averse | 57.89% | 52.63% | 1.000 | 56.52% | 45.83% | 0.564 |
|  |  |  |  |  |  |  |
| Nr. of Subjects | 19 | 19 |  | 23 | 24 |  |
| Notes. Missing data for: ^a^one subject, ^b^two subjects, ^c^one subject. Median GPA for Medical School is 3.5 and for Undergrad studies is 3.7. Entries in the “p-value” columns are for Fisher’s exact test. | | | | | | |
