# Supplementary material for: Effects of patients’ hospital discharge preferences on uptake of clinical decision support
Source: PLoS One. 2021 Mar 8;16(3):e0247270. doi: 10.1371/journal.pone.0247270 (PMC7939268; doi:10.1371/journal.pone.0247270)
Supplement: S6 Appendix — (PDF) [file pone.0247270.s006.pdf]

```
	use Data_PlosOne.dta, clear
******

	label define treatment 1 base 101 baseActor 5 default 103 defaultActor
*drop double recorded data
	sort subjectid patientid period
	drop if subjectid[_n]==subjectid[_n-1] & patientid[_n]==patientid[_n-1] /*
		*/ & period[_n]==period[_n-1]

* selectSubject takes value 1 only once for each subject

	gen selectSubject=1
		replace selectSubject=0 if subjectid[_n]==subjectid[_n+1]

	gen CDS=(treatment==5|treatment==103)

	gen ExpActor=treatment>100

	replace ActorRole=0 if ActorRole==.
		gen Reluctant=ActorRole==2
		gen Eager=ActorRole==1

	tab treatment, gen(dummy)
		rename dummy1 Base
		rename dummy2 Cds
		rename dummy3 BaseActor
		rename dummy4 CdsActor

/****
	Status code: 4 (successful discharge), 2 (unsuccessful discharge)
	Status code: 1 (regular patient), 3 (readmitted patient)
	Recommendation: 1 (Do Not Discharge), 2 (Physician Choice), 3 (Discharge)
****/

	rename Status status

*RR1 records whether patient is readmitted, i.e. the discharge is a success
	gen RR1=status==2
		replace RR1=. if (status==1|status==3)

*choice takes value 1 if the patient is discharged
	gen choice=2-decision

* historical rate of readmissions for patients' procedure codes
	gen targetpr=0.2
		replace targetpr=0.15 if (patientid==18609626|patientid==18734149)
		replace targetpr=0.23 if patientid==23096507
```

```
			replace targetpr=0.17 if patientid==27770106
			replace targetpr=0.14 if patientid==27780512

		gen HighRisk=targetpr>0.17

*survey data risk attitudes:  code all B choices as 1 and A choices as 0
		gen gainrisk=0
		quietly {
			local j=1
			while `j'<11 {
				gen Gain`j'=gain`j'=="B"
					replace gainrisk=`j' if (Gain`j'==1 & gainrisk==0)
			local j=`j'+1
			}
		}
			replace gainrisk=gainrisk-5
		gen RiskAveD=gainrisk>0

/****
		Demographics
****/

		gen Female=gender=="Female"
		gen Athlete=athlete=="Yes"
		gen Musical=musical=="Yes"

* find median gpa (use selection to get one observation per subject)
		tabstat undergradgpa medgpa if selectSubject==1, stats(p50 N)

		gen high_medgpa=medgpa>3.7
		gen high_undergradgpa=undergradgpa>3.5


global demog high_medgpa high_undergradgpa Female Athlete Musical RiskAveD

		egen maxDay=max(periodnumber), by(subjectid)

*Only successful discharges get paid
		gen Earning_Pt=15 if status==4
		egen TotalPay_8Pt=sum(Earning_Pt), by(subjectid)

*Length of Hospital Stay
		gen los1=dayinhospital if choice==1
			replace los1= dayinhospital if choice==0  & periodnumber==maxDay

/******
			Summary Statistics
*****************************************************************************
```

```
*****************************************************************************/

* line 33
	tabstat los1 if choice==1 & ExpActor==1, by(CDS) stats(mean N)
	*need independence of obs for t-test
	egen los1Mean_Subj=mean(los1), by(subjectid)

* line 222-224
	ttest los1Mean_Subj if selectSubject==1 & ExpActor==1, by(CDS)

*line 163: need to count total number of decisions per subject
	gen ChoiceCount=decision
		replace ChoiceCount=decision/2 if decision==2
	egen ChoiceTotal=sum(ChoiceCount), by(subjectid)

	tabstat maxDay ChoiceTot if selectSubj==1 & ExpActor==1, stats(p50 p25 p75 N)

* line 191
	tabstat TotalPay_8Pt if selectSubject==1, by(treatment) stats(mean N)
	ttest TotalPay_8Pt if selectSubject==1 & treatment>100, by(treatment)

/**********
				DATA ANALYSIS
*****************************************************************************/

	tabstat $demog if selectSubject==1, by(treatment) stats(mean N)

/******
	Appendix S4: Demographic Summary Statistics
****/

	foreach var in $demog {
		di _newline _dup(10) "*" as result "( BEGIN of `var' )" _dup(10) "*"
		forval i=1(-1)0 {
			prtest `var' if selectSubject==1 & ExpActor==`i', by(CDS)
			matrix ttest=(r(P1),r(N1),r(P2),r(N2),r(p))
				matrix rownames ttest= `var'`i'
				matrix colnames ttest= Ratio_noCDS N_1 Ratio_CDS N_2 pval
			mat2txt, matrix(ttest) saving("S4_Appendix Demog.xls") append

			tab `var' CDS if selectSubject==1 & ExpActor==`i', col all exact
			matrix Fisher= (r(chi2), r(p), r(p_exact))
				matrix rownames Fisher= `var'`i'
				matrix colnames Fisher= chi2 pval p_exact
			mat2txt, matrix(Fisher) saving("S4_Appendix Demog.xls") append

		}
	}
```

```
* % CDS H:=IF($A2="",".",IF($C2>1,CONCATENATE(ROUND(D2*100,2),"%"),"."))
* % noCDS I:=IF($A2="",".",IF($C2>1,CONCATENATE(ROUND(B2*100,2),"%"),"."))
* Fisher's Exact p J:=IF($A2="",".",IF($C2>1,ROUND(D4,3),"."))

/*************
	Table 1 Length of Hospital Stay
*************/

global out sideway stats(coef ci pval) noaster paren(ci) dec(2) pdec(3) /*
				*/e(r2 r2_p N_clust)

* nr of highrisk patients
	tab patientid HighRisk if period==1 & ExpActor==1
	gen highRisk_cds=HighRisk*CDS

global exo_Var HighRisk CDS highRisk_cds  Reluctant

		reg  los1 startdate $exo_Var if ExpActor==1,  cl(subjectid)
			outreg2  using "Table_1 LOS",  replace excel ct(Actor_1) ti(Reg) $out

		reg  los1 startdate $exo_Var $demog  if ExpActor==1, cl(subjectid)
			outreg2  using "Table_1 LOS",   excel ct(Actor_1) ti(Reg) $out

*censored regression
	gen cnL1=(choice==0 & periodnumber==maxDay)
		replace cnL1=-1 if los1==startdate

	cnreg los1 startdate $exo_Var if ExpActor==1, cl(subjectid) cen(cnL1)
		outreg2  using "cnreg los1",  replace excel ct(Actor_1) ti(Reg) $out

	cnreg los1 startdate $exo_Var $demog if ExpActor==1, cl(subjectid) cen(cnL1)
		outreg2  using "cnreg los1",  excel ct(Actor_1) ti(Reg) $out

/***
	Table 2 Readmissions (Quality of discharge)
***/

	gen LnLos1=ln(los1)

	logit RR1 LnLos1 $exo_Var  if ExpActor==1, cl(subjectid) or
		outreg2 using "Table_2 Readmit", replace eform excel ct(Actor_1) $out

	logit RR1 LnLos1 $exo_Var $demog  if ExpActor==1, cl(subjectid) or
		outreg2 using "Table_2 Readmit", eform excel ct(Actor_1) $out

/*****************************************************************************
*****************************************************************************
```

```
	Table 3 Consistency with CDS
******/

	gen comply=((choice==1 & recommendation==3)|(choice==0 & recommendation==1))
		replace comply=. if recommendation==2

	egen complyMean=mean(comply), by(subjectid ActorRole recommendation)

	sort subjectid ActorRole recommendation periodnumber
		replace complyMean=. if subjectid[_n]==subjectid[_n+1] & /*
	*/recommendation[_n]==recommendation[_n+1] & ActorRole[_n]==ActorRole[_n+1]

/***
	Recommendation: 1 (Do not Discharge) 3 (Discharge)
	Actor Role:	2 (Reluctant)		1 (Eager)
***/

* compliance with "Do Not Discharge" recommendation
* Standardized patient exp. rec=1 is only for the StartDate of one patient
	tab complyMean CDS if rec==1 & ExpActor==1 & ActorR==1, col exact
	tab complyMean CDS if rec==1 & ExpActor==1 & ActorR==2, col exact
	tab complyMean CDS if rec==1 & ExpActor==1, col exact

*line 319
	tabstat complyMean if rec==3 & ExpActor==1, by(CDS) stats(min max N)

	gen Rec=rec+2

	capture program drop Concord
program define Concord
	di _newline _dup(10) "*" as result "( BEGIN of `1' )" _dup(10) "*"
	di _newline as result "CDS is available `2'"
	forval i=5(-2)1 {
		di _newline _dup(5) "." as result "Recommend is `i'" _dup(5) "." /*
					*/ as text " 5(Dis_Rec) 3(DoNot_Rec) 1(All_Rec)" _dup(5) "."
			if `i'>1 {
				ci means complyMean if Rec==`i' & ExpActor==1 & `1'==1 & CDS==`2'
			}
			else {
				ci means complyMean if !(rec==2) & ExpActor==1 & `1'==1 & CDS==`2'
			}
				matrix ci= (r(N), r(mean), r(lb), r(ub))
					matrix rownames ci= `1'_Cds`2'_Rec`i'
					matrix colnames ci= N mean p5 p95
				mat2txt, matrix(ci) saving("Table_3 Concord.xls") append
	}
end
```

```
* Table 3 CDS rows
	foreach var in Reluctant Eager ExpActor {
		Concord `var' 1
	}

*Table 3 no CDS rows
	foreach var in Reluctant Eager ExpActor {
		Concord `var' 0
	}


	capture program drop Concord_ttest
program define Concord_ttest
	di _newline _dup(10) "*" as result "( BEGIN of `1' )" _dup(10) "*"
	forval i=5(-2)1 {
		di _newline _dup(5) "." as result "Recommend is `i'" _dup(5) "." /*
						*/ as text " 5(Dis_Rec) 3(DoNot_Rec) 1(All_Rec)" _dup(5) "."
		if `i'>1 {
			ttest complyMean if Rec==`i' & ExpActor==1 & `1'==1, by(CDS)
		}
		else {
			ttest complyMean if !(rec==2) & ExpActor==1 & `1'==1, by(CDS)
		}
			matrix ttest=(r(mu_1),r(mu_2),r(mu_1)-r(mu_2),r(p))
				matrix rownames ttest= Rec`i'_`1'
				matrix colnames ttest= mean_noCDS mean_CDS mean_diff pval
			mat2txt, matrix(ttest) saving("Table_3 Concord.xls") append
	}
end

*Table 3 Net CDS rows
	foreach var in Reluctant Eager ExpActor {
		Concord_ttest `var'
	}

*mean(95%CI):=IF(A2="",".",IF(B2>2,CONCATENATE(ROUND(C2,2),"  (",ROUND(D2,2),",
",ROUND(E2,2),")"),CONCATENATE(ROUND(D2,2),"   ",ROUND(E2,3))))

/****************************************************************************
****************************************************************************
	Effect of patient preference for discharge on decisions
	Table 4 Estimated Odds Ratio of Discharge
****************/

egen pHigh=pctile(los1) if treatment==1, p(75) by(patientid)
	egen Ready=max(pHigh), by(patientid)
		gen Q4=(dayinhospital>Ready)
```

```
	egen pLow=pctile(los1) if treatment==1, p(25) by(patientid)
	egen NotReady=max(pLow), by(patientid)
		gen Q1=(dayinhospital<NotReady)

global keep keep(1.Reluctant 1.Q4 1.Reluctant#1.Q4 1.Eager 1.Q1 1.Eager#1.Q1)

	logit choice Eager Reluctant CDS, cl(subjectid) or nolog
		outreg2 using "Table_4 Discharge", eform excel ct(all) $out replace

	forvalues i=1(-1)0 {
		logit choice  Reluctant##Q4 i.dayinh $demog if Eager==0 & /*
					*/ CDS==`i', cl(subjectid) or nolog
		outreg2 using "Table_4 Discharge", $keep eform excel ct(Cov_Reluct DS`i') $out
	}

	forvalues i=1(-1)0 {
		logit choice Eager##Q1 i.dayinh $demog if Reluctant==0 & /*
					*/ CDS==`i', cl(subjectid) nolog or
		outreg2 using "Table_4 Discharge", $keep eform excel ct(Cov_Eager DS`i') $out
	}
```
